# Cervical Cancer Biomarkers in Non-Cervical Samples: Emerging Tools for Diagnosis and Prognosis

**DOI:** 10.3390/ijms26136502

**Published:** 2025-07-06

**Authors:** Mélida del Rosario Lizarazo-Taborda, Marisol Godínez-Rubí, Daniel Núnez-Avellaneda, Adrián Ramírez-de-Arellano, Ana Laura Pereira-Suárez, Julio César Villegas-Pineda

**Affiliations:** 1 Instituto de Investigación en Cáncer e Infecciones, Departamento de Microbiología y Patología, Centro Universitario de Ciencias de la Salud, Universidad de Guadalajara, Guadalajara 44340, Mexico; melida.lizarazo1075@alumnos.udg.mx (M.d.R.L.-T.); adrian.ramirez@academicos.udg.mx (A.R.-d.-A.); 2 Laboratorio de Patología Diagnóstica e Inmunohistoquímica, Centro de Investigación y Diagnóstico en Patología, Departamento de Microbiología y Patología, Centro Universitario de Ciencias de la Salud, Universidad de Guadalajara, Guadalajara 44340, Mexico; juliana.godinez@academicos.udg.mx; 3 Dirección Adjunta de Desarrollo Tecnológico, Vinculación e Innovación, Secretaría de Ciencia, Humanidades, Tecnología e Innovación, Ciudad de México 03940, Mexico; daniel.nunez@secihti.mx

**Keywords:** cervical cancer, biomarkers, diagnosis, prognosis, non-cervical samples

## Abstract

Cervical cancer (CC) is the gynecological cancer with the highest incidence and mortality worldwide. High-risk oncogenic human papillomaviruses (HPV) genotypes 16 and 18 are the primary risk factors for developing this female neoplasm, with them being the etiological agents of 70% of cervical cancers. Despite the availability of various prevention strategies, laboratory tests capable of detecting the disease in its previous and early stages, and multiple treatment schemes, CC incidence and mortality rates remain high, due in part to the population’s rejection or disinterest in the current type of sampling. An alternative that could encourage women to take better care of their gynecological health is the availability of tests that detect biomarkers in non-cervical samples with high sensitivity and specificity. The detection of biomarkers in non-cervical samples (blood, serum, plasma, urine, and vaginal fluids) may help reduce the discomfort associated with cervical sampling in patients, therefore promoting gynecological healthcare. This review discusses current diagnostic methods and recent advances in CC biomarkers detected in non-cervical samples, emphasizing their potential for diagnosis, prognosis, and patient monitoring. We further discuss the challenges and future perspectives of applying these biomarkers in clinical practice. The results of this review show that there is a considerable range of biomarkers proposed as alternative tools with high efficacy. Their identification in previous stages of the disease and routinely in non-cervical samples could help reduce the incidence and mortality rates of CC.

## 1. Introduction

**Figure 1:**
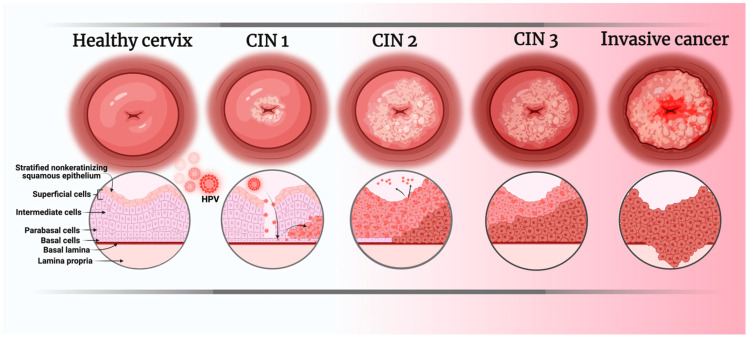
Stages of HPV infection development leading to the appearance of CC. CIN: Cervical intraepithelial neoplasm. Image created in BioRender.com/bszht4w (accessed on 17 June 2025).

Cervical cancer (CC) is the gynecological neoplasia with the highest incidence and mortality worldwide, with 662,301 new cases and 348,874 deaths reported in 2022 [1,2]. The primary risk factor for CC is the human papillomavirus (HPV), which infects immature squamous cells of the transformation zone of the cervix, or the basal cells of the mature squamous epithelium [3] (Figure 1), and disrupts the regulation of cell proliferation by blocking the activity of proteins essential for proper control of cell replication, such as p53 and pRb. Viral proteins E6 and E7 inhibit p53 and Rb, respectively, promoting the development of CC [4,5,6]. HPVs are classified into two major groups based on their oncogenic potential: low-risk and high-risk viruses [7]. The primary viruses responsible for CC are in the high-risk group, with HPV16 and HPV18 being the etiological agents in approximately 70% of cases [8,9].

**Figure 2:**
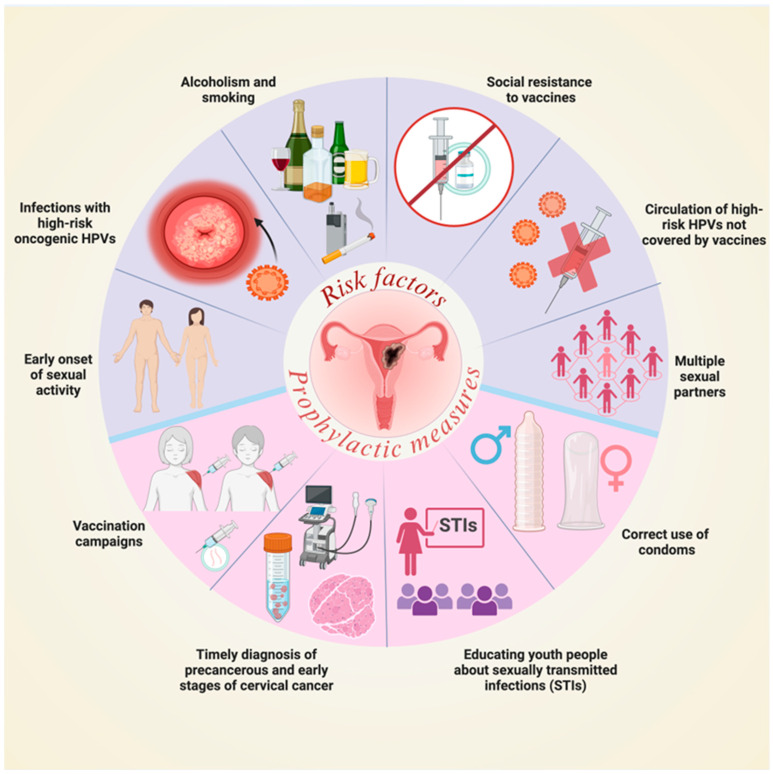
Risk factors for CC and prophylactic measures against HPV infections. STIs: Sexually transmitted infections. Image created in BioRender.com/b71m613 (accessed on 1 July 2025).

Currently, various prophylactic measures exist to prevent HPV infections, including vaccination campaigns against some high-risk oncogenic HPV types, correct condom use, and raising awareness among the youth population about sexually transmitted infections. However, several factors contribute to the persistently high morbidity and mortality rates of CC worldwide. These include early sexual activity, multiple sexual partners, lack of awareness among young people, alcohol consumption, smoking, resistance to vaccination, and the circulation of HPV strains not covered by the vaccine [1,2] (Figure 2).

## 2. Laboratory Tests for the Diagnosis of Cervical Cancer

Currently, there are laboratory tests that are very effective at diagnosing CC, even in its early stages [10,11] (Table 1); these involve visual and tactile examinations, while others require the collection of cells from the cervix to identify abnormalities. Histopathological examinations typically involve biopsies, while molecular tests generally detect the presence of viral nucleic acids in cells collected via cervical brushing [10]. However, the general population does not have the habit of regularly going for gynecological check-ups, probably due to the different degrees of discomfort generated by cervical sampling. Notably, the presence of gynecological pathologies at the time of sampling, such as endometriosis, bleeding, dryness or vaginal atrophy, increased body mass index, musculoskeletal problems, advanced age, or menopause, can result in sample collection being quite painful [12,13]. Additionally, the taboo generated by cultural, religious, social, and demographic aspects also limits women from going to the gynecologist [14].

## 3. Biomarkers in Non-Cervical Samples

Multiple strategies are currently being investigated to identify non-cervical samples that aid in the diagnosis and prognosis of CC, including the use of trained dogs capable of detecting CC-specific volatile organic compounds and discriminating CC from control samples [15], as well as the ongoing search for biomarkers in non-cervical samples [16,17,18,19,20]. Molecular biomarkers in non-cervical samples are emerging as an alternative to cervical sampling to avoid the discomfort, pain, and social taboo generated by cervical sampling performed for current tests. Identification and use of biomarkers in non-cervical samples could help reduce the high incidence and mortality of CC (Table 2).

### 3.1. ncRNAs

#### 3.1.1. miRNAs

Several studies suggest using blood samples to obtain serum or plasma to analyze the presence of microRNAs (miRNAs) [39] since this type of sample causes less discomfort to patients as it only involves venipuncture. Using microRNA array assays, elevated levels of miRNA-1290, miRNA-146a-5p, miRNA-151a-3p, miRNA-2110, and miRNA-21-5p have been found in patients with CC compared to healthy individuals. Due to their high accuracy, these miRNAs are proposed as candidates for the diagnosis of CC [21,22].

A serum miRNA panel consisting of miR-16-2, miR-195, miR-2861, and miR-497 has been proposed to discriminate with high accuracy CC patients from cervical intraepithelial neoplasia (CIN) and healthy subjects. Intriguingly, miRNA-497 has been shown to promote apoptosis through caspase-3/7 activity and also inhibit cell proliferation in HeLa cells, and in a murine model, it was observed that overexpression of miR-497 significantly reduced tumor volume, tumor weight, and tumor formation [23]. Further studies are needed to confirm the pro-tumor role of miR-497 and the clinical relevance of its overexpression in the serum of patients with CC.

To prevent the development of CC, it is very important to diagnose the disease in its early stages [40]. Xin et al. found that miR-9, miR-10a, miR-20a, and miR-196a were upregulated in serum samples derived from patients with CIN compared with serum from healthy controls, and due to their high accuracy, they have suggested that these miRNAs could be useful and novel non-invasive biomarkers for the early detection of CIN [24]. Urine is another type of non-cervical and non-invasive sample that has been proposed as reliable in the early diagnosis of CC. A panel of miRNAs in urine, consisting of miR-145-5p, miR-218-5p, and miR-34a-5p, achieved 100% sensitivity and 92.8% specificity to distinguish precancer and cancer patients from healthy subjects, correlating with the levels found of these miRNAs in serum and tumor tissues. Additionally, miR-34a-5p and miR-218-5p have been proposed as independent prognostic factors for the overall survival of CC patients [25]. The expression characteristics of this panel of miRNAs highlight its usefulness as a diagnostic and prognostic CC biomarker.

The prognosis of cancer patients is crucial for designing the appropriate treatments, planning care, and estimating life expectancy. In this sense, miRNA-142-5p has been proposed as a biomarker of progression and poor clinical outcome, with it being overexpressed in the serum in the late stages of CC (III-IV) compared to the early stages (I-II) of this neoplasia. Evidence indicates that this miRNA is secreted in exosomes by cervical squamous cell carcinoma cells and is internalized by lymphatic endothelial cells. It activates the expression of the enzyme Indoleamine 2,3-dioxygenase (IDO), facilitating the immune escape of cancer cells via CD8+ T cell exhaustion through the conversion of tryptophan to kynurenine. This event is associated with a decrease in tumor-infiltrating lymphocytes (TILs) and poor clinical outcomes in multiple cancers [26].

Biomarkers can also indicate a good prognosis. For example, miRNA-651 is associated with a positive response to cisplatin. It is found at low levels in the plasma of CC patients and at elevated levels in healthy individuals. *In vitro* assays have demonstrated that exosomes loaded with miRNA-651 restrain cisplatin resistance and proliferation and facilitate apoptosis in CC cells. Therefore, miRNA-651 has been proposed as a biomarker of good prognosis and a potential therapeutic agent, capable of improving treatment response by downregulating ATG3 (Autophagy Related 3) [27].

#### 3.1.2. lncRNAs

Long non-coding RNAs (lncRNAs) are another type of ncRNA proposed as diagnostic biomarkers for CC patients [41]. Wang et al. (2020) [28] analyzed the presence of serum lncRNAs in CC and CIN patients and healthy controls. They found that CCAT2, LINC01133, and LINC00511 are highly expressed in the serum of patients with CC, and that, together with the squamous cell carcinoma antigen, they suggest these lncRNAs as effective diagnostic biomarkers. Liu et al. (2023) [42] statistically analyzed a database of 304 CC tumor tissues and three cancer-free tissues. They found that lncRNA AC023043.1 was highly expressed in patients with poor overall survival, while the lncRNAs ZSCAN16-AS1, AC083799.1, AL021707.6, and LINC02356 were associated with a lower risk of death, increased sensitivity to chemotherapy drugs, better overall survival, and better progression-free survival. The authors propose this panel of lncRNAs of prognostic and predictive utility for patients with CC.

### 3.2. RNAs

CC generates differential gene expression even in the early stages of the disease. This has been demonstrated by Sheng and Zhang, who, using oligonucleotide microarrays, analyzed total RNA from peripheral blood lymphocytes. They found significant upregulation of tenasin-c (*TNC*), nucleolin (*NCL*), and enolase 2 (*ENO2*) in the blood of patients with early-stage CC (IB-IIA) compared to healthy controls [29], identifying these RNAs as potential tools for non-invasive diagnosis.

### 3.3. Cell Proteins

Characterizing the tumor protein profile is very important to define the prognosis of patients with CC. Studies conducted with CC cell lines have shown that high-risk HPVs can promote the expression of cellular proteins such as CD55 and CD59 [43]. Both complement regulatory proteins physiologically interfere with the assembly of the membrane attack complex [44,45,46] and have been observed pathologically to generate chemoresistance in patients with breast cancer [47], suggesting that these proteins could potentially be used as prognostic tools for CC patients. Another protein proposed as a diagnostic and prognostic biomarker is tropomyosin 3 (TPM3), which is overexpressed in tumor tissue from CC patients compared to healthy tissue. In both *in vitro* and *in vivo* assays, its presence has been shown to favor cell proliferation, migration, and invasion, as well as tumor growth in animal models. Furthermore, CC patients with high TPM3 expression had poorer overall survival compared to those with low levels of this protein [30]. FBXO5 promotes similar pro-tumor events to those exerted by TPM3, making it another candidate as a dual diagnostic and prognostic biomarker [31].

Other proteins proposed as biomarkers of poor prognosis include cleft lip and palate transmembrane protein 1-like protein (CLPTM1L) and lumican (LUM). Overexpression of either protein in tumor tissue leads to recurrence in CC patients by promoting resistance to chemotherapy, specifically cisplatin. In the particular case of CLPTM1L, this expression is associated with reduced overall survival [32,48]. In addition, it has been shown that chaperonin containing TCP1 subunit 3 (CCT3) expression is involved in the development of CC. This protein is part of the chaperonin-containing TCP1 complex (CCT), which folds various proteins in an ATP-dependent manner [49]. Due to its overexpression in other types of cancers [50,51,52,53], its ability to promote cisplatin resistance [54], and the evidence that CC patients with tumors overexpressing this protein exhibit lower overall survival [55], CCT3 has been proposed as a diagnostic and poor prognostic biomarker.

### 3.4. Biomarkers of Viral Origin

Biomarkers are not necessarily molecules expressed by human cells; there are also biomarkers of viral origin. Specifically, HPV16/18 E7 circulating cell-free DNA (cfDNA) has been proposed as a potential tumor marker with high specificity for minimally invasive CC monitoring. It is differentially expressed in the serum of patients with CC compared to patients with CIN and controls, and it is detected in less than 30 min through sensitive isothermal detection recombinase polymerase amplification combined with a lateral flow strip (RPA-LF) [33]. In pursuit of non-cervical samples, urine has also been proposed as a non-invasive liquid biopsy useful for detecting DNA of viral origin [56]. Lee et al. have proposed a versatile nanowire platform for highly efficient isolation and direct PCR-free colorimetric differential detection of HPV16 and HPV18 DNA from unprocessed urine with high sensitivity and specificity, even when using a small volume of urine (300 µL) [34]. Furthermore, the Cobas^®^ 6800 automated system has been proposed as a next-generation device capable of detecting DNA from HPV16, HPV18, and HPV68 in urine samples [57]. This automated system, based on real-time polymerase chain reaction (PCR), has proven to be highly sensitive (93%, 94%, and 90% for HPV16, HPV18, and HPV68, respectively), 100% specific, and fully reproducible. These characteristics position urine as an excellent non-cervical sample for the detection of HPV.

Other non-cervical samples proposed for the identification of viral DNA are blood samples [18]. It has been shown that HPV16, HPV18, HPV31, HPV33, HPV35, HPV45, HPV52, HPV56, HPV59, HPV58, and HPV73 DNA can be detected in serum and plasma [35,36,37,58] samples from patients with CC, with these DNAs being identified as accurate, non-invasive, inexpensive, and easily accessible biomarkers for the diagnosis, prognosis, therapeutic monitoring and follow-up of patients with CC.

### 3.5. Miscellaneous Biomarkers

In addition to molecules of a biological nature, volatile organic compounds have also been proposed as biomarkers of CC. Rodríguez-Esquivel et al. described the volatolome of the female genitourinary area using Gas Chromatography–Mass Spectrometry. They differentially identified various alkanes with a sensitivity and specificity of 93% in samples from pads used by patients with CC and healthy controls [38]. Another proposed biomarker is the aberrant methylation of Septin9 DNA in plasma. This has shown high discriminatory power in predicting pelvic nodal metastasis of CC, with an optimal specificity of 81.48%. Consequently, aberrant methylation of Septin9 DNA in plasma has been proposed as an innovative and non-invasive biomarker with predictive potential for pelvic lymph node metastasis in CC [59]. Research continues on identifying other biomarkers in non-cervical and non-invasive samples for the diagnosis of CC. The detection of metabolites in plasma [16] and urine [60], free fatty acids in serum [61], and tumor DNA in urine [62] is of considerable interest.

## 4. Characteristics of miRNAs and lncRNAs as Effective Biomarkers of CC in Non-Cervical Samples

As discussed in Section 3, numerous reports highlight the usefulness of biomarkers in non-cervical samples for CC diagnosis, prognosis, prediction, disease monitoring, and patient follow-up, and as shown in Table 2, many of these proposed biomarkers are miRNAs and lncRNAs detected in liquid biopsies (serum, plasma, and urine). The strength of these ncRNAs lies in several qualities they possess: (i) miRNAs/lncRNAs are expressed at disproportionately higher levels compared to other types of biomarkers, such as proteins and mRNAs, since their transcriptional regulatory function requires these elevated expression levels to maintain control of gene expression [63,64]; (ii) they exhibit more tissue- and disease-specific expression patterns, whereas mRNA and protein expression is usually broader and shared among different tissues [65]; (iii) miRNAs/lncRNAs have greater stability in liquid biopsies, as they are usually protected in exosomes or protein complexes [66,67], while mRNAs degrade more easily, a characteristic that makes them less practical for detection in plasma, serum, or urine; (iv) deregulation of miRNAs caused by HPV is evidenced by the fact that miR-15a-5p, miR-17-5p, miR-20a-5p, miR-21-5p, miR-96, miR-106b-5p, and miR-365a are upregulated, while miR-497-5p is downregulated in HPV-infected cell models compared to healthy tissues [68]. Additionally, it has been demonstrated that HPV oncoproteins E6 and E7 can downregulate miRNAs (miR-3156-3p, miR-6779-3p, miR-4779-3p, miR-6811-3p, miR-545-5p, and miR-656-5p) in *in vitro* assays [69] and (v) current research trends highlight ncRNAs as novel and promising elements in various pathologies, particularly cancer [70,71,72,73,74]. These characteristics highlight the utility and relevance of miRNAs and lncRNAs over other types of biomarkers as diagnostic, prognostic, predictive, and monitoring tools in the context of CC.

## 5. Challenges and Future Perspectives of Cervical Cancer Biomarkers in Non-Cervical Samples

The use of CC biomarkers in non-cervical samples—such as blood, plasma, serum, urine, and vaginal fluids—represents a promising alternative to conventional diagnostic methods. Among its main advantages are minimal invasiveness, the possibility of repeated sampling, dynamic disease monitoring, and a more comprehensive assessment of tumor heterogeneity [60,75,76,77]. These features facilitate longitudinal monitoring of patients and can improve early detection, prognosis, and treatment personalization.

The integration of biomarkers into clinical practice faces significant challenges that extend beyond their scientific identification. The transition from discovery to clinical application is a complex and prolonged process that requires close and coordinated collaboration among researchers, clinicians, developers, and statisticians. This multidisciplinary interaction is essential to validate the sensitivity and specificity of biomarkers, yet it is often hindered by reluctance to share data and the need for verification by independent laboratories. Moreover, the high costs associated with advanced technologies, specialized equipment, and clinical trials further complicate widespread implementation, particularly in healthcare systems with limited resources. Collaborative networks such as the Early Detection Research Network (EDRN) promote an integrative model that facilitates access to resources and expertise, thereby accelerating the validation and clinical implementation of biomarkers. However, upon reviewing the EDRN database, a notable absence of reported advances in biomarkers for CC becomes evident, highlighting a significant gap in translational research for this malignancy [78]. This lack of representation may be attributed to the prioritization of other tumors with greater funding and a higher number of validated biomarkers, as well as the availability of effective screening methods for CC, such as cytology and HPV testing. Overcoming these challenges can lead to advancements in the research and development of CC-specific biomarkers, as well as foster multidisciplinary collaboration and investment in clinical validation to overcome the economic and structural barriers that continue to hinder the widespread adoption of these promising tools [79].

Within the clinical implementation of biomarkers in cancer, there are also other significant technical challenges, such as the lack of standardization in collection, processing, and analysis procedures, which limits the reproducibility and comparability of results between different laboratories [57,62,76,80]. Furthermore, the sensitivity and specificity of biomarkers in non-cervical samples have not yet reached the optimal levels required for routine use, which can lead to false-positive or -negative results [35,37,56,57]. Other challenges include the low concentration of biomarkers in peripheral fluids, the need for highly sensitive detection technologies, and the influence of biological and preanalytical factors that can affect the interpretation of results [35,37,56,57,62,76].

Despite the challenges of clinically implementing biomarkers in non-cervical samples, such as the low abundance of tumor genetic material, variability in collection and analysis methods, and the risk of false-positive or false-negative results, advances in ultrasensitive technologies, artificial intelligence, and multi-omics analysis have begun to transform this landscape. In particular, artificial intelligence has become an emerging tool with great potential to improve diagnostic and prognostic accuracy; for example, the use of machine learning algorithms integrated into the analysis of proteomic data derived from mass spectrometry has demonstrated an improved ability to predict CC from serum samples, supporting the identification of discriminating molecular signatures with high sensitivity and specificity [81]. These developments allow for better discrimination between patients and controls, the identification of more robust molecular signatures, and dynamic disease monitoring, which is especially useful for the early detection of recurrences and the evaluation of treatment response [35,37,56,57]. However, to translate these findings into real clinical benefits, it is essential to establish standardized protocols, promote well-designed multicenter studies with external validation, and foster interdisciplinary collaboration between oncologists, laboratory scientists, and biomedical sector stakeholders [18,82,83]. In this sense, although the vast majority of clinical trials related to CC analyze therapeutic regimens [84,85,86], assess the effectiveness of different vaccination schemes [87,88,89], identify biomarkers in cervical samples [90,91,92], promote public awareness regarding cervical screening [93,94,95], and propose imaging studies as preventive and diagnostic tools [96,97,98], the few clinical studies based on CC biomarkers in non-cervical samples are encouraging. 

Clinical studies with CC patients are currently being conducted that (i) measure circulating levels of deoxyribonucleotides, HPV DNA, and tumor cells in blood samples of CC patients treated with radiochemotherapy followed by brachytherapy [99]; (ii) measure high risk HPV DNA in the urine of CIN 2/3 patients [100]; (iii) measure circulating HPV DNA in plasma from women with CIN 1-3 lesions [101]; (iv) measure circulating cell-free tumor tissue modified viral (TTMV)-HPV DNA in blood samples of women with genital dysplasia [102]; and (v) measure aurora kinase A and ninein interacting protein (AUNIP: a protein involved in DNA double-stand break repair) in serum samples of CC patients [103]. These clinical trials are designed to demonstrate the practical utility of these non-cervical samples for diagnosis, prognosis, disease monitoring, and patient follow-up. Among the interventional clinical trials, high-risk HPV DNA and DNA methylation are the main biomarkers analyzed in urine, via multiplex real-time polymerase chain reaction and multiplex methylation-specific quantitative PCR, to establish the diagnosis of CC and precursor lesions. Several trials are being conducted, designed to demonstrate the effectiveness of self-collection of samples for the identification of high-risk HPV DNA [100,104,105,106]. Self-collection of urine samples is ideal due to the favorable perception participants have regarding self-sampling and the non-invasive nature of sample collection [107,108]. The development of clinical trials is important to consolidate the diagnostic and prognostic utility of CC biomarkers in non-cervical samples in the comprehensive management of CC.

## 6. Conclusions

Despite various prophylactic measures against CC, such as vaccination campaigns, information campaigns against HPV infections, and promotion of condom use, among others, this neoplasm remains the leading cause of incidence and mortality among gynecological cancers worldwide. Fortunately, technological innovation in the diagnosis and prognosis of CC is constantly evolving. The detection of molecular biomarkers with high specificity and sensitivity in non-cervical samples emerges as an excellent alternative in the diagnosis and prognosis of CC. Its advantages over classical sampling could favor the reduction in the high incidence (when they are detected in previous stages of CC) and mortality rates of the most frequent gynecological neoplasia worldwide. Non-cervical sampling is a great alternative to cervical sampling because, being minimally invasive or non-invasive, it avoids the pain caused by current sampling, which is uncomfortable in the presence of gynecological morbidities, increased body mass index, musculoskeletal problems, advanced age, or menopause. In addition, it eliminates the taboo generated by cultural, religious, and social aspects that limit women from going to the gynecologist. This type of sampling can improve patient adherence to routine gynecological screening tests.

In conjunction with the hard work carried out in the laboratory, it is necessary that the geographical areas most affected by CC have the infrastructure, economic resources, and personnel necessary to carry out these innovative techniques. Other key strategies include increasing public awareness of sexually transmitted infections, continuing with CC prevention campaigns, and promoting gynecological health.

Biomarkers in non-cervical samples represent accessible tools that can significantly contribute to reducing the incidence and mortality of CC. The biomarkers proposed to date still require routine implementation in laboratory tests for validation and reliable use. Furthermore, the search for new biomarkers must continue to expand the repertoire of molecules that contribute to the diagnosis and prognosis of patients with CC.

## Figures and Tables

**Table 1:** Current tests and procedures for the diagnosis of cervical cancer [10,11].

Common Tests and Procedures	Description
Bimanual pelvic examination	In this study, the physician conducts a manual examination of the uterus, vagina, ovaries, and adjacent organs to identify potential pathological changes. The assessment begins with an inspection of the vulva to detect any visible abnormalities. Subsequently, the physician may insert two fingers of one hand into the vaginal canal while applying gentle pressure to the lower abdomen with the other hand. This bimanual palpation enables the evaluation of internal structures, such as the uterus and ovaries, which are not directly visible during the examination.
Papanicolaou test (cervico-vaginal cytology)	For sample collection, the physician gently obtains exfoliated cells from both the exocervix and endocervix using a soft brush or spatula. The collected cells are then stained using the Papanicolaou (Pap) technique and examined under a microscope to identify cytological abnormalities. These findings may indicate the presence of human papillomavirus (HPV) infection—the main risk factor for CC—or other cellular alterations.
Molecular tests (HPV DNA and mRNA)	If the Papanicolaou test suggests HPV infection, HPV genotyping is performed. Some physicians conduct both tests simultaneously to avoid a second patient visit and to maximize the use of the biological sample. For this examination, cells are collected from the cervix to detect the presence of viral DNA or mRNA, typically from high-risk oncogenic HPV types, which are the primary etiological agents of CC. A positive HPV result does not confirm CC; histopathological examinations from biopsies are required to confirm the cancer diagnosis.
Colposcopy	With the aid of a colposcope—an instrument that provides illumination and magnification for detailed visualization of the cervix—and a sterile speculum to gently distend the vaginal walls, the physician is able to conduct a thorough examination of the cervical epithelium. Colposcopy assists in performing a cervical biopsy by guiding the physician to the suspicious tissue. This procedure is non-invasive, does not cause pain for the patient, and can be performed on pregnant women. Biopsy collection can be guided using two simple diagnostic tests: application of 5% acetic acid or Lugol’s iodine solution (Schiller test), which help to highlight abnormal epithelial areas for targeted sampling.
Biopsy	Histopathological diagnosis is the gold standard for the diagnosis of CC. Biopsy collection involves obtaining a small tissue sample—usually by punch technique—for microscopic analysis by a pathologist. During and after the procedure, the patient may experience cramping, such as menstrual pain, bleeding, and discharge. If the lesion on the cervix is confined to a small area, the physician might remove it entirely during the biopsy (excisional biopsy or conization).

**Table 2:** Novel biomarkers detected in non-cervical samples for the diagnosis, prognosis, and prediction of cervical cancer.

Biomarker Molecule	Sample	Clinical Utility	Technique	Oncological Evidence	References
miRNA-1290	Serum	Diagnostic	Reverse transcription–quantitative polymerase chain reaction (RT-qPCR)	miRNA levels increased in patients with CC compared to healthy patients	[21]
Exosomal miRNA-146a-5p, miRNA-151a-3p, miRNA-2110, and miRNA-21-5p	Plasma	Diagnostic	Reverse transcription–quantitative polymerase chain reaction (RT-qPCR)		[22]
miR-16-2*, miR-195, miR-2861, and miR-497	Serum	Diagnostic	Reverse transcription–quantitative polymerase chain reaction (RT-qPCR)	Panel of miRNAs capable of discriminating CC patients from CIN and healthy subjects	[23]
miR-9, miR-10a, miR-20a, and miR-196a	Serum	Diagnostic	Reverse transcription–quantitative polymerase chain reaction (RT-qPCR)	Panel of useful, novel, and non-invasive miRNAs for the early detection of CIN	[24]
miR-145-5p, miR-218-5p, and miR-34a-5p	Urine and serum	Diagnostic and prognostic	Reverse transcription–quantitative polymerase chain reaction (RT-qPCR)	Panel of miRNAs with 100% sensitivity and 92.8% specificity to distinguish precancer and cancer patients from healthy subjects	[25]
Exosomal miRNA-142-5p	Serum	Progression and poor clinical outcome	Reverse transcription–quantitative polymerase chain reaction (RT-qPCR)	miRNA overexpressed in the late stages of CC (III-IV) compared to the early stages (I-II) of CC	[26]
Exosomal miRNA-651	Plasma	Diagnostic and good prognosis	Reverse transcription–quantitative polymerase chain reaction (RT-qPCR)	miRNA downregulated in cancer subjects compared to healthy individuals, with high sensitivity and accuracy for CC diagnosis	[27]
CCAT2, LINC01133, and LINC00511	Serum	Diagnostic	Reverse transcription–quantitative polymerase chain reaction (RT-qPCR)	lncRNAs are highly expressed in the serum of patients with CC	[28]
*TNC*, *NCL*, and *ENO2*	Blood	Diagnostic	Oligonucleotide microarrays RT-PCR	RNA levels increased in the blood of patients with CC in the early stages (IB-IIA) compared to healthy controls	[29]
TPM3	Tumor tissue	Diagnostic and poor prognosis	Immunohistochemistry Western immunoblotting	Proteins highly expressed in patients with CC and levels increased even more in patients with poor overall survival	[30]
FBXO5	Tumor tissue	Diagnostic and poor prognosis	Immunohistochemistry		[31]
CLPTM1L	Tumor tissue	Predictive and poor prognosis	Immunohistochemistry	Protein overexpression in tumor tissue is associated with recurrence in patients with CC due to its role in promoting resistance to chemotherapy, specifically cisplatin	[32]
HPV16/18 E7 circulating cell-free DNA	Serum	Patient monitoring	Recombinase polymerase amplification–lateral flow (RPA-LF)	cfDNA differentially expressed in the serum of patients with CC compared to patients with CIN and controls	[33]
HPV16 and HPV18 cell-free DNA	Urine	Diagnostic	Spectrophotometry Polyethylenimine-conjugated magnetic nanowires (PEI/mPpy NWs)	cfDNA detected in samples from patients with CC	[34]
cfDNA HPV16, HPV18, HPV33, HPV35, HPV45, HPV56, HPV58, and HPV59	Plasma	Predictive and prognostic	Next-Generation Sequencing (NGS)	Biomarkers potentially useful for monitoring therapy response and detecting relapse	[35]
ctDNA HPV16, HPV18, HPV31, HPV33, HPV45, HPV52, HPV58, and HPV73	Serum and plasma	Predictive and prognostic	Digital droplet PCR (ddPCR)		[36]
ctDNA HPV16 and HPV18	Serum	Predictive and prognostic	Digital droplet PCR (ddPCR)		[37]
Volatile organic compounds (alkanes)	Biofluids from the female genitourinary tract collected on a pad	Diagnostic	Gas Chromatography–Mass Spectrometry	Differential profile of volatile organic compounds between patients with CC and healthy subjects	[38]

## Data Availability

Data is contained within the article.

## References

[1] Bray F., Laversanne M., Sung H., Ferlay J., Siegel R.L., Soerjomataram I., Jemal A. (2024). Global Cancer Statistics 2022: GLOBOCAN Estimates of Incidence and Mortality Worldwide for 36 Cancers in 185 Countries. CA Cancer J. Clin..

[2] Ferlay J., Ervik M., Lam F., Laversanne M., Colombet M., Mery L., Piñeros M., Znaor A., Soerjomataram I., Bray F. (2024). Global Cancer Observatory: Cancer Today, (Version 1.1).

[3] Woodman C.B.J., Collins S.I., Young L.S. (2007). The Natural History of Cervical HPV Infection: Unresolved Issues. Nat. Rev. Cancer.

[4] Thomas M., Pim D., Banks L. (1999). The Role of the E6-P53 Interaction in the Molecular Pathogenesis of HPV. Oncogene.

[5] Ramakrishnan S., Partricia S., Mathan G. (2015). Overview of High-Risk HPV’s 16 and 18 Infected Cervical Cancer: Pathogenesis to Prevention. Biomed. Pharmacother..

[6] Vats A., Trejo-Cerro O., Massimi P., Banks L. (2022). Regulation of HPV E7 Stability by E6-Associated Protein (E6AP). J. Virol..

[7] An H.J., Cho N.H., Lee S.Y., Kim I.H., Lee C., Kim S.J., Mun M.S., Kim S.H., Jeong J.K. (2003). Correlation of Cervical Carcinoma and Precancerous Lesions with Human Papillomavirus (HPV) Genotypes Detected with the HPV DNA Chip Microarray Method. Cancer.

[8] Mayo T.T., Imtiaz R., Doan H.Q., Sambrano B.L., Gordon R., Ramirez-Fort M.K., Tyring S.K. (2014). Human Papillomavirus: Epidemiology and Clinical Features of Related Cancer. Viruses and Human Cancer.

[9] Burd E.M. (2003). Human Papillomavirus and Cervical Cancer. Clin. Microbiol. Rev..

[10] American Society of Clinical Oncology (ASCO) Cervical Cancer: Diagnosis. https://www.cancer.net/cancer-types/cervical-cancer/diagnosis.

[11] National Cancer Institute (NCI) Cervical Cancer Diagnosis. https://www.cancer.gov/types/cervical/diagnosis.

[12] Landy R., Hollingworth T., Waller J., Marlow L.A., Rigney J., Round T., Sasieni P.D., Lim A.W. (2022). Non-Speculum Sampling Approaches for Cervical Screening in Older Women: Randomised Controlled Trial. Br. J. Gen. Pr..

[13] Ørnskov D., Jochumsen K., Steiner P.H., Grunnet I.M., Lykkebo A.W., Waldstrøm M. (2021). Clinical Performance and Acceptability of Self-Collected Vaginal and Urine Samples Compared with Clinician-Taken Cervical Samples for HPV Testing among Women Referred for Colposcopy. A Cross-Sectional Study. BMJ Open.

[14] Afsah Y.R., Kaneko N. (2023). Barriers to Cervical Cancer Screening Faced by Immigrant Muslim Women: A Systematic Scoping Review. BMC Public Health.

[15] Guerrero-Flores H., Apresa-García T., Garay-Villar Ó., Sánchez-Pérez A., Flores-Villegas D., Bandera-Calderón A., García-Palacios R., Rojas-Sánchez T., Romero-Morelos P., Sánchez-Albor V. (2017). A Non-Invasive Tool for Detecting Cervical Cancer Odor by Trained Scent Dogs. BMC Cancer.

[16] Khan I., Nam M., Kwon M., Seo S., Jung S., Han J.S., Hwang G.-S., Kim M.K. (2019). LC/MS-Based Polar Metabolite Profiling Identified Unique Biomarker Signatures for Cervical Cancer and Cervical Intraepithelial Neoplasia Using Global and Targeted Metabolomics. Cancers.

[17] Nakabayashi M., Kawashima A., Yasuhara R., Hayakawa Y., Miyamoto S., Iizuka C., Sekizawa A. (2018). Massively Parallel Sequencing of Cell-Free DNA in Plasma for Detecting Gynaecological Tumour-Associated Copy Number Alteration. Sci. Rep..

[18] Andrioaie I.M., Luchian I., Dămian C., Nichitean G., Andrese E.P., Pantilimonescu T.F., Trandabăț B., Prisacariu L.J., Budală D.G., Dimitriu D.C. (2023). The Clinical Utility of Circulating HPV DNA Biomarker in Oropharyngeal, Cervical, Anal, and Skin HPV-Related Cancers: A Review. Pathogens.

[19] Lee S.-Y., Chae D.-K., Lee S.-H., Lim Y., An J., Chae C.H., Kim B.C., Bhak J., Bolser D., Cho D.-H. (2020). Efficient Mutation Screening for Cervical Cancers from Circulating Tumor DNA in Blood. BMC Cancer.

[20] Ning R., Meng S., Wang L., Jia Y., Tang F., Sun H., Zhang Z., Zhang C., Fan X., Xiao B. (2021). 6 Circulating MiRNAs Can Be Used as Non-Invasive Biomarkers for the Detection of Cervical Lesions. J. Cancer.

[21] Nagamitsu Y., NishiI H., Sasaki T., Takaesu Y., Terauchi F., Isaka K. (2016). Profiling Analysis of Circulating MicroRNA Expression in Cervical Cancer. Mol. Clin. Oncol..

[22] Ma G., Song G., Zou X., Shan X., Liu Q., Xia T., Zhou X., Zhu W. (2019). Circulating Plasma MicroRNA Signature for the Diagnosis of Cervical Cancer. Cancer Biomark..

[23] Zhang Y., Zhang D., Wang F., Xu D., Guo Y., Cui W. (2015). Serum MiRNAs Panel (MiR-16-2*, MiR-195, MiR-2861, MiR-497) as Novel Non-Invasive Biomarkers for Detection of Cervical Cancer. Sci. Rep..

[24] Xin F., Liu P., Ma C.-F. (2016). A Circulating Serum MiRNA Panel as Early Detection Biomarkers of Cervical Intraepithelial Neoplasia. Eur. Rev. Med. Pharmacol. Sci..

[25] Aftab M., Poojary S.S., Seshan V., Kumar S., Agarwal P., Tandon S., Zutshi V., Das B.C. (2021). Urine miRNA Signature as a Potential Non-Invasive Diagnostic and Prognostic Biomarker in Cervical Cancer. Sci. Rep..

[26] Zhou C., Zhang Y., Yan R., Huang L., Mellor A.L., Yang Y., Chen X., Wei W., Wu X., Yu L. (2021). Exosome-Derived MiR-142-5p Remodels Lymphatic Vessels and Induces IDO to Promote Immune Privilege in the Tumour Microenvironment. Cell Death Differ..

[27] Zhu X., Long L., Xiao H., He X. (2021). Cancer-Derived Exosomal MiR-651 as a Diagnostic Marker Restrains Cisplatin Resistance and Directly Targets ATG3 for Cervical Cancer. Dis. Markers.

[28] Wang W.-J., Wang D., Zhao M., Sun X.-J., Li Y., Lin H., Che Y.-Q., Huang C.-Z. (2020). Serum LncRNAs (CCAT2, LINC01133, LINC00511) with Squamous Cell Carcinoma Antigen Panel as Novel Non-Invasive Biomarkers for Detection of Cervical Squamous Carcinoma. Cancer Manag. Res..

[29] Sheng J., Zhang W. (2010). Identification of Biomarkers for Cervical Cancer in Peripheral Blood Lymphocytes Using Oligonucleotide Microarrays. Chin. Med. J..

[30] Zhao Y.-C., Wang T.-J., Qu G.-H., She L.-Z., Cui J., Zhang R.-F., Qu H.-D. (2023). TPM3: A Novel Prognostic Biomarker of Cervical Cancer That Correlates with Immune Infiltration and Promotes Malignant Behavior In Vivo and In Vitro. Am. J. Cancer Res..

[31] Jiang S., Zheng J., Cui Z., Li Y., Wu Q., Cai X., Zheng C., Sun Y. (2023). FBXO5 Acts as a Novel Prognostic Biomarker for Patients with Cervical Cancer. Front. Cell Dev. Biol..

[32] Awazu Y., Fukuda T., Noda T., Uchikura E., Nanno S., Imai K., Yamauchi M., Yasui T., Sumi T. (2023). CLPTM1L Expression Predicts Recurrence of Patients with Intermediate- and High-risk Stage IB-IIB Cervical Cancer Undergoing Radical Hysterectomy Followed by TP as Adjuvant Chemotherapy. Oncol. Lett..

[33] Rungkamoltip P., Temisak S., Piboonprai K., Japrung D., Thangsunan P., Chanpanitkitchot S., Chaowawanit W., Chandeying N., Tangjitgamol S., Iempridee T. (2021). Rapid and Ultrasensitive Detection of Circulating Human Papillomavirus E7 Cell-Free DNA as a Cervical Cancer Biomarker. Exp. Biol. Med..

[34] Lee H., Choi M., Hwang S.-H., Cho Y. (2018). A Versatile Nanowire Platform for Highly Efficient Isolation and Direct PCR-Free Colorimetric Detection of Human Papillomavirus DNA from Unprocessed Urine. Theranostics.

[35] Mittelstadt S., Kelemen O., Admard J., Gschwind A., Koch A., Wörz S., Oberlechner E., Engler T., Bonzheim I., Staebler A. (2023). Detection of Circulating Cell-Free HPV DNA of 13 HPV Types for Patients with Cervical Cancer as Potential Biomarker to Monitor Therapy Response and to Detect Relapse. Br. J. Cancer.

[36] Cabel L., Bonneau C., Bernard-Tessier A., Héquet D., Tran-Perennou C., Bataillon G., Rouzier R., Féron J.-G., Fourchotte V., Le Brun J.-F. (2021). HPV CtDNA Detection of High-Risk HPV Types during Chemoradiotherapy for Locally Advanced Cervical Cancer. ESMO Open.

[37] Jeannot E., Latouche A., Bonneau C., Calméjane M.-A., Beaufort C., Ruigrok-Ritstier K., Bataillon G., Chérif L.L., Dupain C., Lecerf C. (2021). Circulating HPV DNA as a Marker for Early Detection of Relapse in Patients with Cervical Cancer. Clin. Cancer Res..

[38] Rodríguez-Esquivel M., Rosales J., Castro R., Apresa-García T., Garay Ó., Romero-Morelos P., Marrero-Rodríguez D., Taniguchi-Ponciano K., López-Romero R., Guerrero-Flores H. (2018). Volatolome of the Female Genitourinary Area: Toward the Metabolome of Cervical Cancer. Arch. Med. Res..

[39] do Nascimento Medeiros J.A., Sarmento A.C.A., Bernardes-Oliveira E., de Oliveira R., Lima M.E.G.B., Gonçalves A.K., de Souza Dantas D., de Oliveira Crispim J.C. (2023). Evaluation of Exosomal MiRNA as Potential Biomarkers in Cervical Cancer. Epigenomes.

[40] Zheng M., Hou L., Ma Y., Zhou L., Wang F., Cheng B., Wang W., Lu B., Liu P., Lu W. (2019). Exosomal Let-7d-3p and MiR-30d-5p as Diagnostic Biomarkers for Non-Invasive Screening of Cervical Cancer and Its Precursors. Mol. Cancer.

[41] Iempridee T., Wiwithaphon S., Piboonprai K., Pratedrat P., Khumkhrong P., Japrung D., Temisak S., Laiwejpithaya S., Chaopotong P., Dharakul T. (2018). Identification of Reference Genes for Circulating Long Noncoding RNA Analysis in Serum of Cervical Cancer Patients. FEBS Open Bio.

[42] Liu L., Liu J., Lyu Q., Huang J., Chen Y., Feng C., Liu Y., Chen F., Wang Z. (2023). Disulfidptosis-Associated LncRNAs Index Predicts Prognosis and Chemotherapy Drugs Sensitivity in Cervical Cancer. Sci. Rep..

[43] Khan A., Hussain S., Iyer J.K., Kaul A., Bonnewitz M., Kaul R. (2023). Human Papillomavirus-Mediated Expression of Complement Regulatory Proteins in Human Cervical Cancer Cells. Eur. J. Obs. Gynecol. Reprod. Biol..

[44] Detsika M.G., Palamaris K., Dimopoulou I., Kotanidou A., Orfanos S.E. (2024). The Complement Cascade in Lung Injury and Disease. Respir. Res..

[45] Lukacik P., Roversi P., White J., Esser D., Smith G.P., Billington J., Williams P.A., Rudd P.M., Wormald M.R., Harvey D.J. (2004). Complement Regulation at the Molecular Level: The Structure of Decay-Accelerating Factor. Proc. Natl. Acad. Sci. USA.

[46] Couves E.C., Gardner S., Voisin T.B., Bickel J.K., Stansfeld P.J., Tate E.W., Bubeck D. (2023). Structural Basis for Membrane Attack Complex Inhibition by CD59. Nat. Commun..

[47] Montalvo-Castro R.E., Salinas-Jazmín N. (2022). Relationship between the Expression of Complement Inhibitory Proteins and Therapeutic Efficacy of Antibodies in Breast Cancer. Gac. Med. Mex..

[48] Hu G., Xiao Y., Ma C., Wang J., Qian X., Wu X., Zhu F., Sun S., Qian J. (2023). Lumican Is a Potential Predictor on the Efficacy of Concurrent Chemoradiotherapy in Cervical Squamous Cell Carcinoma. Heliyon.

[49] National Library of Medicine (NLM) (2024). CCT3 Chaperonin Containing TCP1 Subunit 3 [Homo Sapiens (Human)], Gene ID: 7203.

[50] Wang K., He J., Tu C., Xu H., Zhang X., Lv Y., Song C. (2022). Upregulation of CCT3 Predicts Poor Prognosis and Promotes Cell Proliferation via Inhibition of Ferroptosis and Activation of AKT Signaling in Lung Adenocarcinoma. BMC Mol. Cell Biol..

[51] Wang Y., Liu P., Zhang Z., Wang J., Cheng Z., Fan C. (2021). Identification of CCT3 as a Prognostic Factor and Correlates with Cell Survival and Invasion of Head and Neck Squamous Cell Carcinoma. Biosci. Rep..

[52] Qian T., Cui L., Liu Y., Cheng Z., Quan L., Zeng T., Huang W., Dai Y., Chen J., Liu L. (2020). High Expression of Chaperonin-Containing TCP1 Subunit 3 May Induce Dismal Prognosis in Multiple Myeloma. Pharmgenom. J..

[53] Cui X. (2015). Overexpression of Chaperonin Containing TCP1, Subunit 3 Predicts Poor Prognosis in Hepatocellular Carcinoma. World J. Gastroenterol..

[54] Danni X., Jiangzheng Z., Huamao S., Yinglian P., Changcheng Y., Yanda L. (2021). Chaperonin Containing TCP1 Subunit 3 (CCT3) Promotes Cisplatin Resistance of Lung Adenocarcinoma Cells through Targeting the Janus Kinase 2/Signal Transducers and Activators of Transcription 3 (JAK2/STAT3) Pathway. Bioengineered.

[55] Li M., Zeng J., Chang Y., Lv L., Ye G. (2023). CCT3 as a Diagnostic and Prognostic Biomarker in Cervical Cancer. Crit. Rev. Eukaryot. Gene Expr..

[56] Téblick L., Van Keer S., De Smet A., Van Damme P., Laeremans M., Cortes A.R., Beyers K., Vankerckhoven V., Matheeussen V., Mandersloot R. (2021). Impact of Collection Volume and DNA Extraction Method on the Detection of Biomarkers and HPV DNA in First-Void Urine. Molecules.

[57] Hajjar B., Raheel U., Manina R., Simpson J., Irfan M., Waheed Y. (2023). Clinical Performance of Cobas 6800 for the Detection of High-Risk Human Papillomavirus in Urine Samples. Vaccines.

[58] Galati L., Combes J.-D., Le Calvez-Kelm F., McKay-Chopin S., Forey N., Ratel M., McKay J., Waterboer T., Schroeder L., Clifford G. (2022). Detection of Circulating HPV16 DNA as a Biomarker for Cervical Cancer by a Bead-Based HPV Genotyping Assay. Microbiol. Spectr..

[59] Bu Q., Luo X., He L., Ma J., He S., Lei W., Zhou W., Deng H., Lin Y., Zhang L. (2023). Septin9 DNA Methylation as a Promising Biomarker for Cervical Cancer. J. Obstet. Gynaecol..

[60] Cao Y., Qin Y., Cheng Q., Zhong J., Han B., Li Y. (2025). Bifunctional Nanomaterial Enabled High-Specific Isolation of Urinary Exosomes for Cervical Cancer Metabolomics Analysis and Biomarker Discovery. Talanta.

[61] Katoh Y., Kubo A., Hayashi N., Sugi T., Katoh K., Udagawa S., Ogawa T., Iwata T., Nishio H., Sugawara M. (2024). Serum Levels of Stearic and Dihomo-γ-Linolenic Acids Can Be Used to Diagnose Cervical Cancer and Cervical Intraepithelial Neoplasia. Sci. Rep..

[62] Hentschel A.E., van den Helder R., van Trommel N.E., van Splunter A.P., van Boerdonk R.A.A., van Gent M.D.J.M., Nieuwenhuijzen J.A., Steenbergen R.D.M. (2021). The Origin of Tumor DNA in Urine of Urogenital Cancer Patients: Local Shedding and Transrenal Excretion. Cancers.

[63] Bartel D.P. (2004). MicroRNAs. Cell.

[64] Esteller M. (2011). Non-Coding RNAs in Human Disease. Nat. Rev. Genet..

[65] Ludwig N., Leidinger P., Becker K., Backes C., Fehlmann T., Pallasch C., Rheinheimer S., Meder B., Stähler C., Meese E. (2016). Distribution of MiRNA Expression across Human Tissues. Nucleic Acids Res..

[66] Dai J., Su Y., Zhong S., Cong L., Liu B., Yang J., Tao Y., He Z., Chen C., Jiang Y. (2020). Exosomes: Key Players in Cancer and Potential Therapeutic Strategy. Signal Transduct. Target. Ther..

[67] Mitchell P.S., Parkin R.K., Kroh E.M., Fritz B.R., Wyman S.K., Pogosova-Agadjanyan E.L., Peterson A., Noteboom J., O’Briant K.C., Allen A. (2008). Circulating MicroRNAs as Stable Blood-Based Markers for Cancer Detection. Proc. Natl. Acad. Sci. USA.

[68] Gao D., Zhang Y., Zhu M., Liu S., Wang X. (2016). MiRNA Expression Profiles of HPV-Infected Patients with Cervical Cancer in the Uyghur Population in China. PLoS ONE.

[69] Xia Y.-F., Pei G.-H., Wang N., Che Y.-C., Yu F.-S., Yin F.-F., Liu H.-X., Luo B., Wang Y.-K. (2017). MiR-3156-3p Is Downregulated in HPV-Positive Cervical Cancer and Performs as a Tumor-Suppressive MiRNA. Virol. J..

[70] Slack F.J., Chinnaiyan A.M. (2019). The Role of Non-Coding RNAs in Oncology. Cell.

[71] Brillante S., Volpe M., Indrieri A. (2024). Advances in MicroRNA Therapeutics: From Preclinical to Clinical Studies. Hum. Gene Ther..

[72] Bayraktar E., Bayraktar R., Oztatlici H., Lopez-Berestein G., Amero P., Rodriguez-Aguayo C. (2023). Targeting MiRNAs and Other Non-Coding RNAs as a Therapeutic Approach: An Update. Noncoding RNA.

[73] Shi Y., Liu Z., Lin Q., Luo Q., Cen Y., Li J., Fang X., Gong C. (2021). MiRNAs and Cancer: Key Link in Diagnosis and Therapy. Genes.

[74] Rupaimoole R., Slack F.J. (2017). MicroRNA Therapeutics: Towards a New Era for the Management of Cancer and Other Diseases. Nat. Rev. Drug Discov..

[75] Armakolas A., Kotsari M., Koskinas J. (2023). Liquid Biopsies, Novel Approaches and Future Directions. Cancers.

[76] Ma L., Guo H., Zhao Y., Liu Z., Wang C., Bu J., Sun T., Wei J. (2024). Liquid Biopsy in Cancer: Current Status, Challenges and Future Prospects. Signal Transduct. Target. Ther..

[77] Heidrich I., Ačkar L., Mossahebi Mohammadi P., Pantel K. (2021). Liquid Biopsies: Potential and Challenges. Int. J. Cancer.

[78] National Cancer Institute (NCI) Early Detection Research Network. https://edrn.nci.nih.gov/.

[79] Wagner P.D., Srivastava S. (2012). New Paradigms in Translational Science Research in Cancer Biomarkers. Transl. Res..

[80] Weaver C., Nam A., Settle C., Overton M., Giddens M., Richardson K.P., Piver R., Mysona D.P., Rungruang B., Ghamande S. (2024). Serum Proteomic Signatures in Cervical Cancer: Current Status and Future Directions. Cancers.

[81] Zhang D., Zhao L., Guo B., Guo A., Ding J., Tong D., Wang B., Zhou Z. (2025). Integrated Machine Learning Algorithms-Enhanced Predication for Cervical Cancer from Mass Spectrometry-Based Proteomics Data. Bioengineering.

[82] Kori M., Arga K.Y. (2018). Potential Biomarkers and Therapeutic Targets in Cervical Cancer: Insights from the Meta-Analysis of Transcriptomics Data within Network Biomedicine Perspective. PLoS ONE.

[83] Martinelli C., Ercoli A., Vizzielli G., Burk S.R., Cuomo M., Satasiya V., Kacem H., Braccia S., Mazzarotti G., Miriello I. (2025). Liquid Biopsy in Gynecological Cancers: A Translational Framework from Molecular Insights to Precision Oncology and Clinical Practice. J. Exp. Clin. Cancer Res..

[84] Fracasso P.M., Duska L.R., Thaker P.H., Gao F., Zoberi I., Dehdashti F., Siegel B.A., Uliel L., Menias C.O., Rehm P.K. (2022). An Exploratory Study of Neoadjuvant Cetuximab Followed by Cetuximab and Chemoradiotherapy in Women With Newly Diagnosed Locally Advanced Cervical Cancer. Am. J. Clin. Oncol..

[85] Obstetrics & Gynecology Hospital of Fudan University Iparomlimab and Tuvonralimab Combined with Paclitaxel and Cisplatin as Neoadjuvant Therapy for CC (ClinicalTrials.gov Identifier: NCT06878222). NCT06878222.

[86] Women’s Hospital School Of Medicine Zhejiang University A Clinical Study Comparing Chemotherapy Combined with PD-1 Inhibitor Versus Concurrent Chemoradiotherapy in Cervical Cancer Patients with Positive Lymph Nodes After Surgery: A Multicenter Randomized Controlled Clinical Trial (ClinicalTrials.gov Identifier: NCT06866951). NCT06866951.

[87] Wu Q., Qian M., Welby S., Guignard A., Rosillon D., Gopala K., Xu Y., Liu K., He Y., Jiang N. (2023). Prospective, Multi-Center Post-Marketing Surveillance Cohort Study to Monitor the Safety of the Human Papillomavirus-16/18 AS04-Adjuvanted Vaccine in Chinese Girls and Women Aged 9 to 45 Years, 2018–2020. Hum. Vaccines Immunother..

[88] Charles University, Czech Republic The Adjuvant Effect of HPV Vaccination on Recurrence of Cervical Precancer or Carcinoma in Women Undergoing Conization (ClinicalTrials.gov Identifier: NCT06258564). NCT06258564.

[89] Salmerón J., Torres-Ibarra L., Bosch F.X., Cuzick J., Lörincz A., Wheeler C.M., Castle P.E., Robles C., Lazcano-Ponce E. (2016). HPV vaccination impact on a cervical cancer screening program: Methods of the FASTER-Tlalpan Study in Mexico. Salud Publica Mex..

[90] Chao T.K., Ke F.Y., Liao Y.P., Wang H.C., Yu C.P., Lai H.C. (2013). Triage of cervical cytological diagnoses of atypical squamous cells by DNA methylation of paired boxed gene 1 (PAX1). Diagn. Cytopathol..

[91] Mackay Memorial Hospital The Biomarker Analysis in Locally Advanced (ClinicalTrials.gov Identifier: NCT03635216). NCT03635216.

[92] Wuhan University Molecular Markers in Cervical Cancer Screening in the Feasibility of the Mathematical Markov Model Analysis (ClinicalTrials.gov Identifier: NCT00889902). NCT00889902.

[93] International Agency for Research on Cancer Effectiveness and Impact of the Lived Experience Cancer Awareness Campaign on Screening Participation (ClinicalTrials.gov Identifier: NCT06874985). NCT06874985.

[94] Gül Öztaş H., Işik K. (2025). The Effect of Cervical Cancer Education Given to Women in Turkey on Knowledge, Attitudes, and Health Beliefs: A Randomized Controlled Study. Public Health Nurs..

[95] Istanbul University—Cerrahpasa Web-Based Education Program and Educational Booklet Developed on Cervical Cancer (ClinicalTrials.gov Identifier: NCT06705309). NCT06705309.

[96] Hunt B., Fregnani J.H.T.G., Brenes D., Schwarz R.A., Salcedo M.P., Possati-Resende J.C., Antoniazzi M., de Oliveira Fonseca B., Santana I.V.V., de Macêdo Matsushita G. (2021). Cervical lesion assessment using real-time microendoscopy image analysis in Brazil: The CLARA study. Int. J. Cancer.

[97] Brookdale University Hospital Medical Center Diagnostic Imaging Aid for Management of Cervical Lesions (FFC) (ClinicalTrials.gov Identifier: NCT02406365). NCT02406365.

[98] Aaron J., Nitin N., Travis K., Kumar S., Collier T., Park S.Y., José-Yacamán M., Coghlan L., Follen M., Richards-Kortum R. (2007). Plasmon resonance coupling of metal nanoparticles for molecular imaging of carcinogenesis in vivo. J. Biomed. Opt..

[99] Fabian D. Clinical Trial of Molecular Biomarkers in Women with Uterine Cervix Cancer (ClinicalTrials.gov Identifier: NCT05462951). NCT05462951.

[100] Universiteit Antwerpen First-Void Urine Samples for the Follow-Up of Women Treated for High-Grade Cervical Intraepithelial Neoplasia (CIN) (ClinicalTrials.gov Identifier: NCT03542513). NCT03542513.

[101] UNC Lineberger Comprehensive Cancer Center Application of Plasma Circulating HPV DNA Testing to Management of Cervical Intraepithelial Neoplasia (ClinicalTrials.gov Identifier: NCT04274465). NCT04274465.

[102] University of Mississippi Medical Center Clinical Translational and Biomarker-Based Female Genital HPV Induced Dysplasia and Cancer Screening Study Using Cf-HPV-DNA Blood Tests (TTMV HPV DNA) (ClinicalTrials.gov Identifier: NCT05536843). NCT05536843.

[103] Yuan L. Study on AUNIP as a Novel Tumor Marker for Cervical Cancer (ClinicalTrials.gov Identifier: NCT06118463). NCT06118463.

[104] Universiteit Antwerpen Dry Run of the ScreenUrSelf Trial (ClinicalTrials.gov Identifier: NCT05996796). NCT05996796.

[105] Hendrickx J.O., Van Keer S., Donders G., Weyers S., Doyen J., Beyers K.C.L., Rios-Cortes A., Meers N., Téblick L., Vankerckhoven V.V.J. (2025). Home-based urinary HPV self-sampling for the detection of cervical cancer precursor lesions: Attitudes and preferences from Belgian females participating in the CASUS study. Arch. Public Health.

[106] Universiteit Antwerpen Developing a Combined Molecular Screening and Triage Test for Cervical Cancer in Self-Samples (COMBISCREEN) (ClinicalTrials.gov Identifier: NCT06598176). NCT06598176.

[107] Universiteit Antwerpen Cervical Cancer Screening Based on First-Void Urine Self-Sampling to Reach Un(Der)-Screened Women: ScreenUrSelf Trial (ScreenUrSelf) (ClinicalTrials.gov Identifier: NCT05996783). NCT05996783.

[108] Buelens C., Stabel M., Wildiers A., Peremans L., Van Hal G., Van Doorsselaere L., Lievens A., Vorsters A., Van Keer S., Verhoeven V. (2024). Experiences and Perceptions of Cervical Cancer Screening Using Self-Sampling among Under-Screened Women in Flanders. Healthcare.

